# Substantial changes in the depth distributions of benthic invertebrates in the eastern Kattegat since the 1880s

**DOI:** 10.1002/ece3.4395

**Published:** 2018-08-29

**Authors:** Alf B. Josefson, Lars‐Ove Loo, Mats Blomqvist, Johan Rolandsson

**Affiliations:** ^1^ Department of Bioscience Aarhus University Roskilde Denmark; ^2^ Department of Marine Sciences – Tjärnö University of Gothenburg Strömstad Sweden; ^3^ Hafok AB Stenhamra Sweden; ^4^ EcoWorks AB Ellös Sweden

**Keywords:** bottom trawling, chronic fishing disturbance, elements of metacommunity structure analysis, eutrophication, long‐term change in community structure, marine invertebrates, Scandinavian seas, water depth distribution

## Abstract

Bottom trawling and eutrophication are well known for their impacts on the marine benthic environment in the last decades. Evaluating the effects of these pressures is often restricted to contemporary benthic data, limiting the potential to observe change from an earlier (preimpact) state. In this study, we compared benthic species records from 1884 to 1886 by CGJ Petersen with recent data to investigate how benthic invertebrate species in the eastern Kattegat have changed since preimpact time. The study shows that species turnover between old and recent times was high, ca. 50%, and the species richness in the investigation area was either unchanged or higher in recent times, suggesting no net loss of species. Elements of metacommunity structure analysis of datasets from the 1880s, 1990s, and 2000s revealed a clear change in the depth distribution structure since the 1880s. The system changed from a *Quasi‐nested/Random* pattern unrelated to depth in the 1880s with many species depth ranges over a major part of the studied depth interval, to a *Clementsian* pattern in recent times strongly positively correlated with depth. Around 30% of the 117 species recorded both in old and in recent times, including most trawling‐sensitive species, that is large, semiemergent species, showed a decrease in maximal depth of occurrence from the deeper zone fished today to the shallower unfished zone, with on average 20 m. Concurrently, the species category remaining in the fished zone was dominated by species less sensitive to bottom trawling like infauna polychaetes and small‐sized *Peracarida* crustaceans, most likely with short longevity. The depth interval and magnitude of the changes in depth distribution and the changes in species composition indicate impacts from bottom trawling rather than eutrophication. Furthermore, the high similarity of results from the recent datasets 10 years apart suggests chronic impact keeping the system in an altered state.

## 1 INTRODUCTION

Almost no areas globally are now unaffected by humans, and many areas, including the Scandinavian seas, are regarded highly impacted by several pressures (Halpern et al., 2008, 2015). Therefore, a frequent problem when evaluating human environmental impact is the paucity of good contemporary reference data. Two major recent threats to the marine fauna and flora in Scandinavian coastal marine environments are eutrophication and fishery impacts due to bottom trawling, recognized in the recent 3–4 decades. Here we take advantage of a temporal reference, the benthic invertebrate records from the surveys with the canon ship “Hauch,” performed in the 1880s in the Kattegat by CGJ Petersen (1893). This is the earliest comprehensive survey dataset on benthic invertebrate fauna available from the Scandinavian Sea area, from a time before major impact of the mentioned threats. The surveys took place in the beginning of the industrial expansion in the Scandinavian countries, before the rational agriculture and the application of water closets, and before intensive use of bottom trawls, which required strong motor‐driven vessels. Effective trawling therefore increased sharply first in the 1930s with the introduction of diesel engines (Lindeboom & de Groot, 1998).

Several previous studies have used benthic historic data to set reference conditions in the Scandinavian Sea area. Stephenson, Williams, and Cook (1972) reanalyzed Petersen data from the Danish waters taken some 25 years later than the data from Petersen (1893), and the binary approach mainly confirmed the broad categorization into “Petersen communities.” Revisits of historic sites in the 1970s and 1980s focusing on biomasses reported some support for organic enrichment of the area. Cederwall and Elmgren (1980) visited sites in the Baltic Sea, Pearson, Josefson, and Rosenberg (1985) sites in the Danish sector of the Kattegat established in 1911–1912 (Petersen, 1913), and Rosenberg, Gray, Josefson, and Pearson (1987) sites in the Skagerrak and Oslofjord from 1914 (Petersen, 1915). As the Petersen investigations in 1911–1914 had the purpose to determine the amount of fish food, the taxonomic resolution was much lower than in the study in the 1880s. For instance, polychaete species were often lumped into one category, “vermes.” To our knowledge, our study is the first one using historical data to examine changes in spatial distributions of species in the Kattegat in relation to different environmental threats.

Effects of eutrophication have been obvious in the Scandinavian sea areas during the second half of the twentieth century, an impact now showing signs of reversal after peaking in the 1980s (Andersen et al., 2017; Carstensen, Conley, Andersen, & Ærtebjerg, 2006; Riemann et al., 2016). Concurrently, there are reports of decreased Secchi depths in recent times (e.g., Middelboe & Sand‐Jensen, 2000), which, at least partly, may be related to eutrophication. This together with reports on deeper distribution of eelgrass (*Zostera marina*) in the late 1800s (Petersen, 1893) than in the second half of the 1900s (e.g., Duarte, 1991) indicates a narrowing of the primary productive euphotic zone, with potential effects on benthic micro‐ and macroalgal consumers like grazing snails and some filtering bivalves. Another effect of eutrophication, predominant in some stagnant deeper areas, is the promotion of hypoxia/anoxia, causing mortality of benthic invertebrates (Diaz & Rosenberg, 2008).

Several fishery activities have impacts on marine ecosystems (Dayton, Thrush, Agardy, & Hofman, 1995). While impact due to overfishing, particularly removal of large top predators, has occurred for a very long time (Jackson et al., 2001), bottom trawling is a more recent phenomenon and poses a particular threat to benthic habitats (Thrush & Dayton, 2002). In addition to direct injury of benthic organisms, benthic trawling may homogenize the benthic environment and thereby reduce the habitat complexity at several scales, with adverse effects on biodiversity (Hewitt, Thrush, Halliday, & Duffy, 2005; Thrush & Dayton, 2002; Thrush et al., 1998; Watling & Norse, 1998). Several recent studies from the northeastern Atlantic area report adverse effects of trawling on the benthic fauna such as reduced biodiversity, biomass, and production (Hiddink et al., 2006; Kaiser & Spencer, 1996; Sköld et al., 2018; Van Denderen, Hintzen, Rijnsdorp, Ruardij, & van Kooten, 2014). Trawling may also affect species trait composition by causing a shift from communities dominated by relatively sessile, emergent, high biomass species to communities dominated by small‐bodied infauna (Kaiser, Ramsay, Richardson, Spence, & Brand, 2000). In the area of the present study, effects of trawling are likely depth dependent because intense trawling activities nowadays mainly take place at water depths greater than ca. 30 m (ICES, 2016; Pommer, Olesen, & Hansen, 2016).

**Figure 1:**
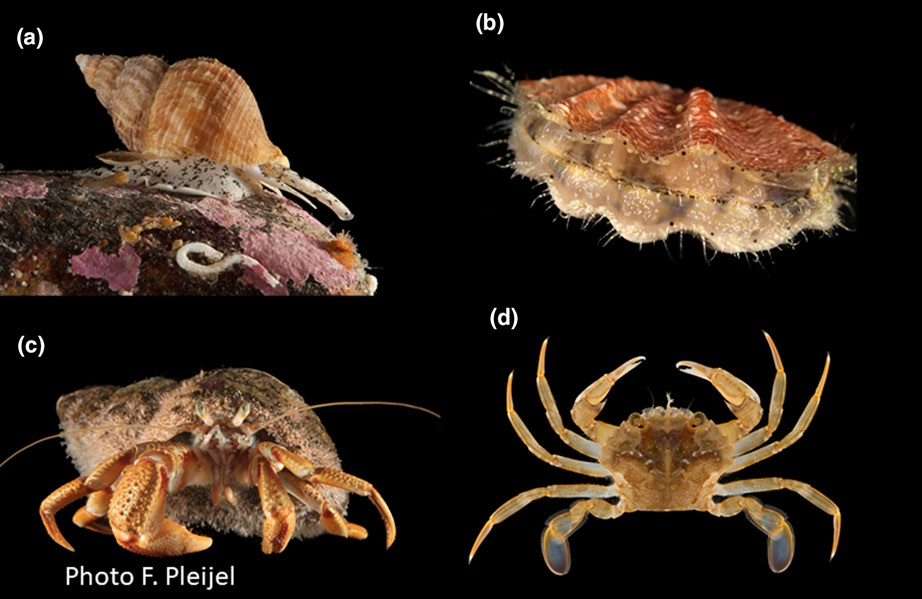
Examples of large emergent species from the investigation area sensitive to bottom trawling: (a) the gastropod *Buccinum*, (b) the pectinid bivalve *Pseudamussium*, only recorded from the 1880s, (c) the eremite crab *Pagurus*, and (d) the crab *Liocarcinus*

Because potential effects of the mentioned threats are depth dependent, our main hypothesis is that the depth distribution structure of the benthic species has changed since preimpact time in the 1880s. We apply the elements of metacommunity structure (EMS) approach (Leibold & Mikkelson, 2002), an unbiased method where the data determine the pattern. The method has proven effective to identify the distribution pattern along the main gradients in several realms (Heino, Soininen, Alahuhta, Lappalainen, & Virtanen, 2017; Josefson, 2016; Meynard et al., 2013; Presley, Higgins, & Willig, 2010; Presley & Willig, 2010; Valanko, Heino, Westerbom, Viitasalo, & Norkko, 2015). We expected trawling‐sensitive species (Figure 1) to disappear or occur only in nonfished areas away from the trawled zone if trawling impact was important, leaving richness in trawled areas dominated by trawling‐insensitive species.

## 2 MATERIALS AND METHODS

### 2.1 Sampling

**Figure 2:**
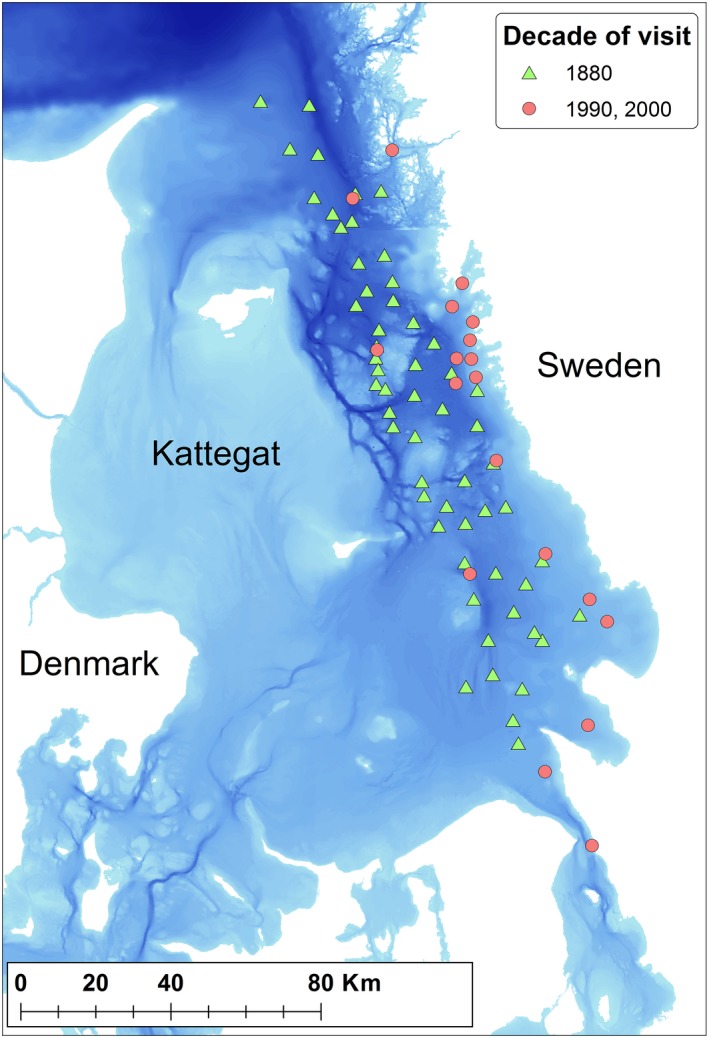
Map over the investigated area with sampling sites. Darkening of the blue color denotes increasing water depth

We compare the water depth distribution structure and species richness in the 1880s dataset with datasets from the 1990s and the 2000s in the same subarea (i.e., the Swedish sector of the Kattegat) at sites with similar bottom type and in the same depth interval, that is 19–80 m (Figure 2, Supporting Information Table S1).

*The 1880s*: The 1880 dataset is qualitative (binary) with the aim to describe the spatial distribution of the common species in the area (Petersen, 1893). Consequently, the 1880 data from the whole Kattegat have a high spatial (a total of >500 sites) and a high taxonomic resolution (>570 species).

We selected 57 sites in the depth interval of 19–80 m from the Swedish sector of Kattegat for study, and the bottom substrate was at least partly fine sediments like sand, silt, or mud (Figure 2, Supporting Information Table S1). We determined positions of sampling sites ourselves from bearings and distance from fixed points like lighthouses and water depths given in fathoms (Petersen, 1893), seemingly in fair agreement with recent bathymetrical maps. Sampling in the 1880s used both trawls and dredges, mostly simultaneously at the same site. There is no information on mesh sizes of gears or of eventual sieves for extraction, but figures given on body sizes of a few millimeters of many mollusk species and records of many small‐bodied crustaceans like most cumaceans indicate a high degree of detail in the examination of the samples (Petersen, 1893). The surveys took place in the four sequential years 1883–1886.

*The 1990s and 2000s*: The same 19 soft sediment sites in the water depth interval of 19–80 m (Figure 2, Supporting Information Table S1) were visited in the years 1998, 1999 and in 2008, 2009, and sampled with five Van Veen grab samples at each site in each year. As each grab covered a bottom area of 0.1 m^2^, the sample from each site in each of the decades 1990s and 2000s covered 1 m^2^. Extraction of fauna from these samples was performed using 1‐mm sieve and otherwise followed standard procedures in the Baltic Sea area (HELCOM, 1988).

### 2.2 Data treatment and approach

Taxonomic identities were compared and adjusted using the species list WorMS http://www.marinespecies.org/.

We applied community analyses on the full datasets from each decade. Additionally, in order to evaluate whether the depth distribution of the same individual species had changed over the >100 year period, analyses were run on the subset of species recorded both in old and in recent times (Supporting Information Tables S2 and S3).

In order to evaluate the effects of bottom trawling, we examined changes in individual species occurrences in two depth zones: one shallower than 30 m (19–30 m) with low fishing pressure (LFP) and one deeper than 30 m (30–80 m) with high pressure (HFP) during the last decades. We based the division of the depth gradient into zones with different fishing pressures on fishing abrasion data from ICES (ICES, 2016).

### 2.3 Elements of metacommunity structure analysis

The elements of metacommunity structure (EMS) approach uses a stepwise procedure and can simultaneously test for multiple idealized patterns across a set of sites (Leibold & Mikkelson, 2002). First, we ordered the site‐by‐species incidence matrices, with sites a priori sorted by depth, with reciprocal averaging (RA) in correspondence analysis (CA). Then, objective criteria based on coherence, turnover, and boundary clumping were used to assess the correspondence of the empirical data set with each of the hypothetical idealizations of species distribution, that is checkerboard, nested, evenly spaced, *Gleasonian*, or *Clementsian* patterns (Leibold & Mikkelson, 2002; Presley et al., 2010; Valanko et al., 2015). The significance of the index value for coherence and turnover was tested using a fixed‐proportional null model, which maintains species richness of each site (i.e., row sums are fixed), but species ranges are filled based on their marginal probabilities (i.e., the “r1” null model, Gotelli, 2000; Dallas, 2014). We used 1,000 simulations to provide random matrices, with zero rows allowed in the null matrices because of sparse measured site‐by‐species matrices (Dallas, 2014). The observed index values were compared to the distribution of index values derived from randomization with the null model to assess statistical significance. We interpreted the results of the EMS analysis according to Presley et al. (2010) and used the metacommunity function in the “metacom” package (version 1.5.0, 2018‐01‐20) for calculations and the “Image” function for graphs of sorted matrices (Dallas, 2014), in the R environment (R Core Team R version 3.4.3, 2017‐11‐30). The data used for the EMS analysis were binary, that is presence–absence of species. Pearson r correlation was used to test whether latent main environmental gradient (i.e., primary axis site scores from RA in correspondence analysis) was significantly correlated with the measured predictor variable (i.e., water depth).

### 2.4 Test of potential bias due to different number of sites and different distributions of site depths

The 1880s dataset contained more sampling sites and a higher proportion of deep sites than the recent datasets (Supporting Information Table S1). To evaluate a potential bias of the EMS results and total richness, we therefore permuted station sets in the old dataset with the same number of sites (19) as in the recent datasets, and for each permutation constructed new site‐by‐species matrices subsequently subjected to EMS analysis in order to determine the depth structure pattern. We applied Pearson r correlation analysis between the site scores on the main RA axis and depth for each permutation. This allowed us to estimate the likelihood of the same pattern in the 1880s using 19 sites as in recent times. The permutations, 600 times, were performed both on the 1800 dataset with all species and the set with species occurring both in old and in recent times.

### 2.5 Fishing pressure

We used benthic abrasion data from ICES (ICES, 2016) to assess fishing pressure from bottom trawling. Values of surface abrasion for the period 2009–2015 were matched with positions of the 19 sites visited in recent times. Abrasion values, that is the SAR—swept area ratio, are the accumulative area in a quadrate swept by trawls in 1 year divided by the quadrate area (Eigaard et al., 2017; ICES, 2016). The SARs were constructed from satellite tracking of fishing vessels longer than 12 m. We assume that the trawling intensity in the period 2009–2015 is representative for at least two preceding decades in the study area, supported by lack of significant correlation between fishing abrasion values in year 1 versus year 7 across all sites (Pearson *r* = 0.038, *p* = 0.665, *n* = 7).

## 3 RESULTS

### 3.1 Fishing pressure

**Figure 3:**
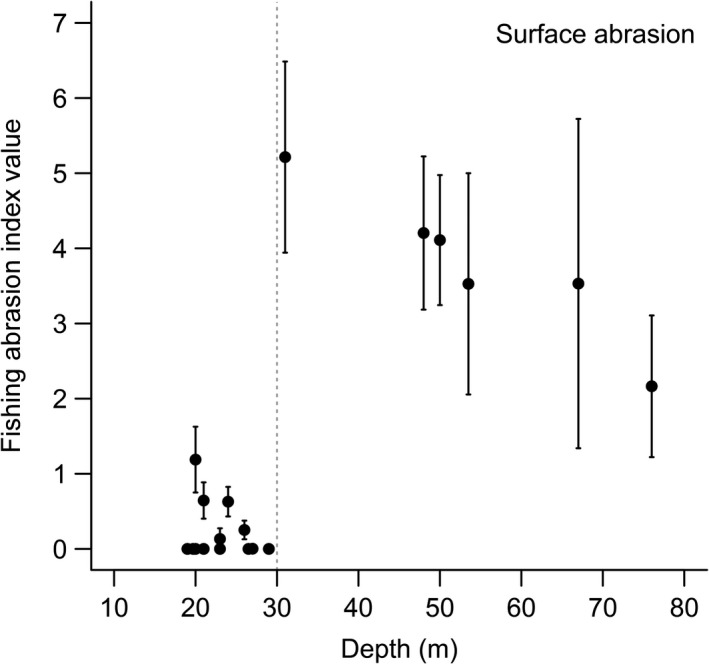
Fishing pressure values, averages of surface abrasion values ±*SD* for the period 2009–2015, versus depth at the sites visited in the 1990s and 2000s. Satellite tracking of fishing vessels longer than 12 m yielded pressure values (ICES, 2016)

Fishing pressure due to bottom trawling in the period 2009–2015 was clearly higher at sites deeper than 30 m than at shallower sites (Figure 3), with the highest pressure immediately below the 30 m depth.

### 3.2 Community changes

The total number of species from the three decades was similar: 239 in the 1880s, 236 in the 1990s, and 241 in the 2000s. Of these, only ca. half—117 species—were recorded both from the 1880s and from one or both of the two recent decades (Supporting Information Table S2). Thus, species turnover between old and recent times was ca. 50%.

**Figure 4:**
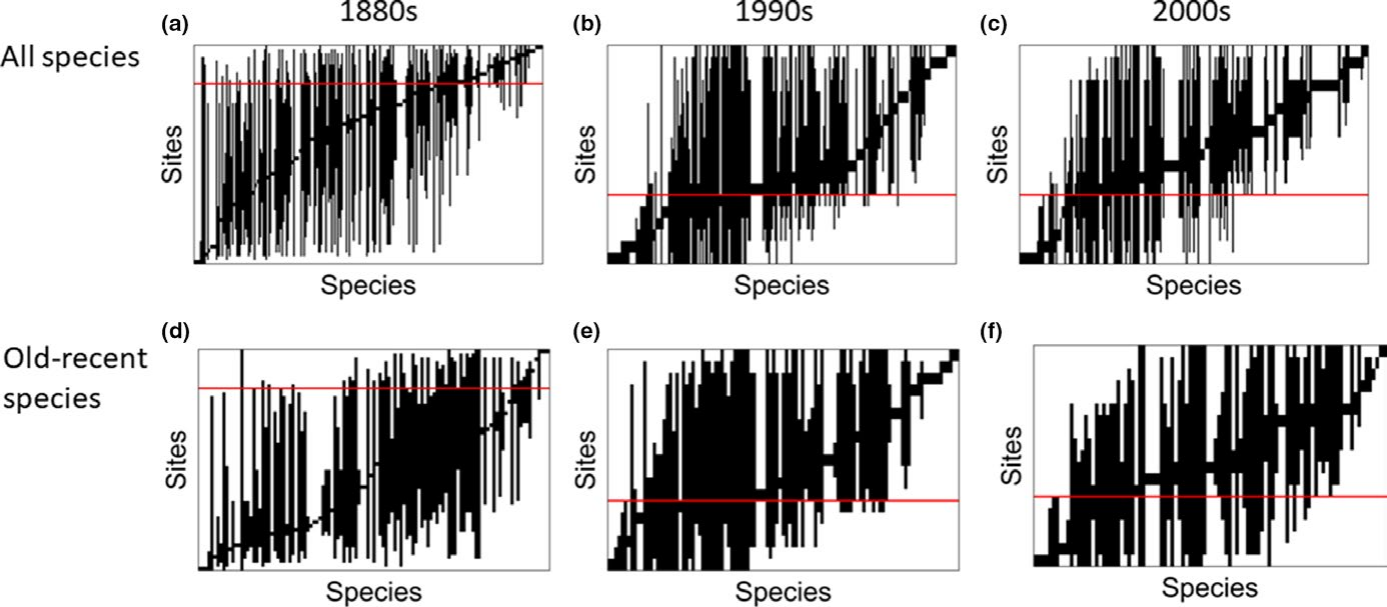
Site‐by‐species matrices of all species (panel a–c) and of species occurring both in old and in recent times (117 species, panel d–f) with sites on *y*‐axis sorted by increasing depth from the top and species on *x*‐axis ordinated by reciprocal averaging. Vertical columns denote species site ranges, that is with absences embedded. Horizontal red line indicates 30 m depth

**Figure 5:**
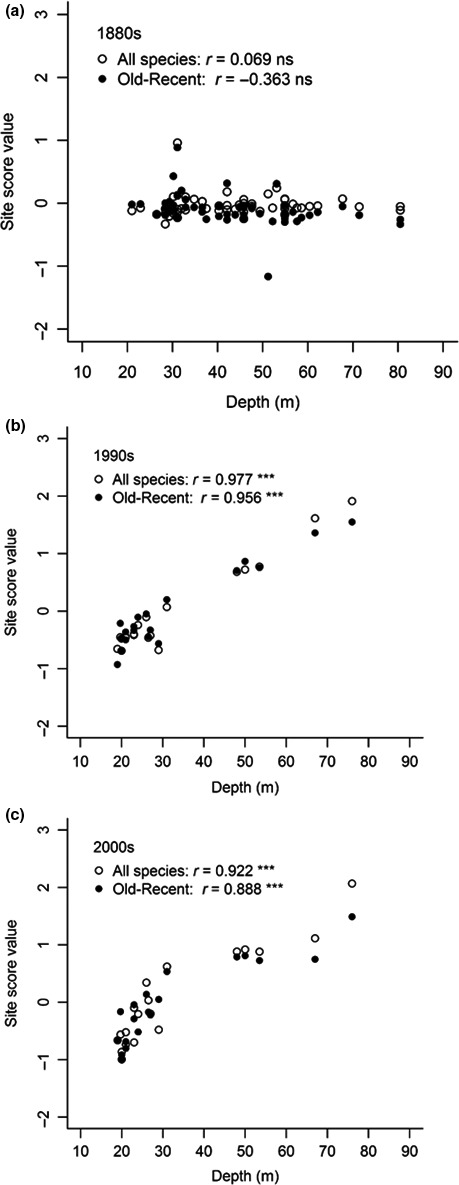
Plots of site scores on the primary RA axis versus water depth from (a) the 1880s, (b) the 1990s, and (c) 2000s. “Old–recent” denotes species present both in the 1880s and in recent times. *r* = Pearson product–moment correlation coefficient, ns = *p* > 0.05, ***p < 0.001. One outlier excluded in the graph from the 1880s

Using all species, there were highly significant coherent patterns in all three decades, that is fewer embedded absences than predicted by the null model (Table 1). Turnover, the number of replacements, was higher than predicted from the model but insignificant in the 1880s, while turnover was significantly higher than expected in the two recent decades (Table 1). Clumping of ranges was significantly higher in both recent decades. Thus, we classified the structure in the 1880s as *Quasi‐nested*, which means that most species had completely overlapping depth distributions with many intervals reaching over the whole studied depth range and that species depth ranges were close to randomly distributed along the main RA axis (Figure 4a). In contrast, we classified the states in the 1990s and the 2000s as *Clementsian*, which means that many species had narrower depth distribution intervals partly overlapping and with groups of species with similar ranges ordered along the main RA axis (Figure 4b,c). Site scores on this axis were correlated strongly and positively with water depth (Figure 5).

**Table 1:** Results of EMS analysis including all species

Decade	Coherence				Species turnover				Boundary clumping		
	Absences	*p*	Mean	*SD*	Replacements	*p*	Mean	*SD*	Morisita's index	*p*	*df*
1880	8,095	<0.001	9,411	16.9	866,030	0.190	787,106	245	1.73	<0.001	54
1990	1,753	<0.001	2,501	9.24	148,935	<0.001	110,407	86.4	1.27	<0.001	16
2000	1,861	<0.001	2,661	10.1	146,810	0.016	128,242	87.8	1.60	<0.001	16

*Note*

Degrees of freedom (*df*) = number of sites—3. Mean and *SD* obtained from the null model.

Results of the same analysis using only the species recorded both in old and in recent times were similar to the results using all species (Figure 4d–f, Table 2). The only difference was that the 1880s dataset did not show significant coherence and was therefore indistinguishable from random.

**Table 2:** Results of EMS analysis including only species present both in old and in recent times

Decade	Coherence				Species turnover				Boundary clumping		
	Absences	*p*	Mean	*SD*	Replacements	*p*	Mean	*SD*	Morisita's index	*p*	*df*
1880	4,410	0.225	4,208	12.9	270,627	0.108	230,036	159	1.46	<0.001	54
1990	681	<0.001	991	6.51	31,252	<0.001	22,643	51.0	1.47	<0.001	16
2000	704	<0.001	894	6.43	29,039	<0.001	21,236	48.5	1.27	<0.001	16

*Note*

Degrees of freedom (*df*) = number of sites—3. Mean and *SD* obtained from the null model.

While the site scores on the primary RA axis were virtually unrelated to water depth in the 1880s, the scores from the two recent patterns were highly significantly (*p* < 0.001) positively correlated with water depth (Figure 5).

### 3.3 Test of potential bias due to different number of sites and different distributions of site depths

To test the robustness of the difference in depth structure between old and recent times, we permuted station sets in the old dataset with the same number of sites (19) as in the recent datasets, and for each permutation repeated the EMS analysis (Table 3). Around 30% of the permutations give a *Clementsian* pattern (183/600), whereas this is 50% (303/600) for species occurring both in old and in recent times. The probability of a *Clementsian* pattern and a significant relationship between site scores and depth is 0.057 (34/600) and 0.058 (35/600), respectively, for the two species categories, whereas a similarly strong relationship as in recent times only happens with a probability of 0.005 (3/600).

**Table 3:** Results of permutation test of potential bias due to different number of sites and different proportions of deep and shallow sites in the 1880s and recent samplings

Selection sampled	No. of permutations	No. of permutations giving *Clementsian* pattern	Permutations with significant positive Pearson *r* (*p* < 0.001) between site scores and depth		p of recent pattern with Pearson *r* > 0.80
			No	*p* of recent pattern	
1880s data—all species	600	183	34	0.057	0.005
1880s data—only species occurring both in old and in recent times	600	303	35	0.058	0.005

*Note*

Each permutation in the old data sets with the site number used in the recent samplings (19) generated a site‐by‐species matrix analyzed with EMS for distribution pattern. Pearson r is the product–moment correlation coefficient between site scores on the main RA axis and depth.

### 3.4 Change in depth distribution of individual species occurring both in old and in recent times

**Figure 6:**
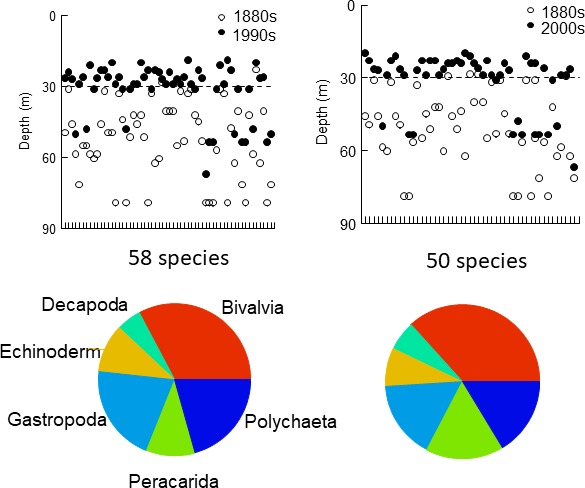
Upper panels: Maximal depths of occurrence for species (*x*‐axes) showing decreasing maximal depth between old and recent times. Broken lines indicate 30 m depth. Lower limit of the depth window was set to 79 m. Pie charts describe percentage composition in terms of higher taxonomic groups

The maximal depth of occurrence, that is the lower depth limit of the depth range, decreased for 58 species in the 1990s and 50 species in the 2000s with on average ca. 20 m for most species up to, or above, 30 m depth (Table 4, Figure 6). This species category included species from all six main taxonomic groups, and more than half of the species belonged to *Bivalvia*,* Gastropoda*, and *Decapoda*.

**Table 4:** Results of two‐group Student's *t* test of species depth records (in meters) from two dates, 1880s versus 1990s and 1880s versus 2000s

Species category	1880s vs. 1990s				
	*df*	Mean 1880s	Mean 1990s	*t*	*p*
Decreasing max depth	57	52.2	31.6	11.93	<0.001
Increasing max depth	40	47.3	67.3	−9.02	<0.001

	1880s vs. 2000s				
	*df*	Mean 1880s	Mean 2000s	*t*	*p*
Decreasing max depth	49	50.9	31.7	10.48	<0.001
Increasing max depth	35	45.7	68.4	−9.1	<0.001

*Note*

*df* = number of species—1. Max depth = Maximal depth of occurrence.

**Figure 7:**
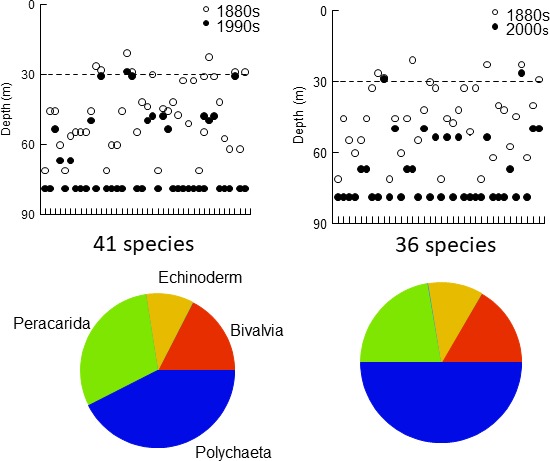
Upper panels: Maximal depths of occurrence for species (*x*‐axes) showing increasing maximal depth between old and recent times. More information in legend of Figure 6

Concurrently, 41 species in the 1990s and 36 in the 2000s, in the 1880s mainly found in the HFP zone, increased their maximal depth of occurrence with at least ca. 20 m, many to the greatest depth of the study (Table 4, Figure 7). The maximal depth limits of remaining species, 10 species in 1990s and 11 species in 2000s, were unchanged relative to the 1880s. While the decreasing group contained species from six higher groups, the increasing group contained species from four groups (no gastropods or decapod crustaceans at all), and 74% of the species belonged to *Polychaeta* and *Peracarida* crustaceans.

### 3.5 Change with respect to traits with different sensitivities to bottom trawling

**Figure 8:**
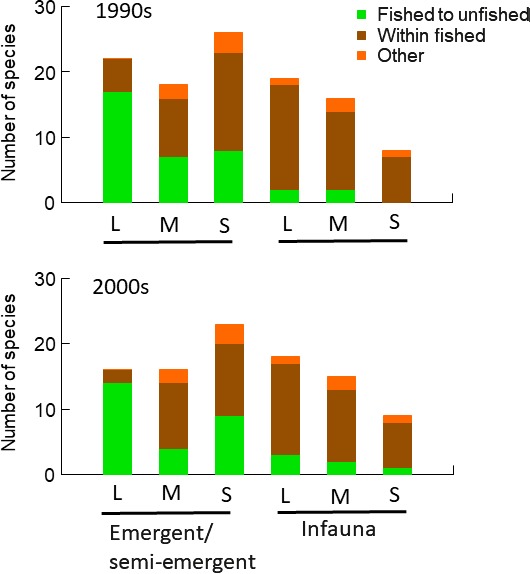
Species distributed over trait categories based on maximal body size: >5 cm (L), 1–5 cm (M) and <1 cm (S), and position in relation to the sediment–water interface: emergent/semiemergent from the sediment and infauna with bodies mostly buried in the sediment. Colors indicate the presence of maximal depth of occurrence in, and changes between, the two depth zones with different fishing pressures: <30 m = unfished (LFP) and >30 m = fished (HFP) zones in recent times. Green color indicates change in maximal depth from the HFP zone in the 1880s to the LFP zone in recent times. Brown indicates that maximal depth remained in the HFP zone in both old and recent times. Orange indicates both species that moved maximal depth from the LFP zone in the 1880s to the HFP zone in recent times, as well as species with their maximal depth remaining in the LFP zone in both old and recent times

We prepared Supporting Information Table S3 with all species occurring both in old and in recent times and their maximal depth occurrence as well as assignments of traits with different sensitivities to trawling and indications of type of change in the two zones with different fishing pressures. We summarize the results for the two important traits body size and degree of emergence from the sediment in Figure 8.

Very few species had their deepest occurrence in the unfished LFP zone in both old and recent times, 3.7% and 5.2% in the 1990s and 2000s, respectively, and few species had changed maximal depth occurrence from the LFP zone to the fished HFP zone, 4.6% and 4.1% in the 1990s and 2000s, respectively. Almost 60% of the species in all periods remained within the HFP zone, and many of them have a deeper occurrence in recent times (Figure 7). In contrast, 33% of the species both in the 1990s and in the 2000s have moved their maximal depth of occurrence from the HFP zone in the 1800s to the LFP zone in recent times. In fact, this included most large (>5 cm) emergent or semiemergent species, while most large infauna and burrowing species remained in the HFP zone (Figure 8).

Generally, much more emergent species than infauna species and more large than small species have moved their maximal depth of occurrence from the fished HFP zone to the unfished LFP zone in recent times (Figure 8).

The depth distribution structure was similar in the 1990s and 2000s (Figure 4). The comparisons between the 1880s and the two recent datasets were similar with respect to magnitude of changes and the taxonomic and trait composition. Together, this suggests little change over the recent 10‐year period (Figures 4, 5, 6, 7, 8, Table 4).

### 3.6 Species richness

Richness is dependent on sampling effort, and because, for example, the number of sites differs greatly between the old and recent datasets, this precludes direct comparison between the datasets. Therefore, using the rarefaction in the above‐described permutation test, we estimated richness in 19‐site samples in the old datasets for comparison with the recent datasets (Table 5). Richness in the 1880s estimated from 19‐site samples was considerably lower than measured richness in the recent datasets, which were well above the upper limit of the estimated mean + 1SD (Table 5). Much of this difference, however, was due to a higher fraction of polychaete species in recent datasets, 44%–48% compared to 18% in the 1880s. Assuming that the same fractions of polychaetes and nonpolychaetes in the estimated mean richness as in the measured richness from 1880s indicate similar level of richness of the nonpolychaete fauna in old and recent times, richness of polychaetes increased threefold in recent times (Table 5).

**Table 5:** Estimated richness (S) at 19 sites from 600 permutations in the 1880s dataset and measured richness in the three datasets

	1880s Estimated S ± *SD* 19 sites	1880s Measured S 57 sites	1990s Measured S 19 sites	2000s Measured S 19 sites
All species	159.6 ± 11.8	239	236	241
Polychaete species	28.1	42	103	115
Non‐polychaete species	132	197	133	126

## 4 DISCUSSION

The different sampling gears in old and recent times makes quantitative comparisons between periods problematic. The dredges and trawls used in the 1880s likely sampled large, epifaunal organisms with low density much more efficiently than the grabs used in recent times, while the grabs used in recent times probably sampled abundant Infauna such as polychaetes more efficiently. Nor can we exclude some taxonomic turnover because of different investigators. These conditions could have contributed to the modest overlap (ca. 50%) in species composition between old and recent times. Indeed, infauna polychaetes are much more specious in the recent datasets. Part of the high richness of polychaetes in recent times may also be due to taxonomic evolution. Species from several genera like *Nepthys, Pholoe*,* Chaetopterus* has been split into more species rather than merged into fewer species, and several new polychaete species have been described later than the surveys in 1880s (e.g., Hartmann‐Schröder, 1996). To overcome some of these methodological biases, we, in addition to using binary data only, also compared distributions between time periods of those species actually recorded both in old and in recent times. For the depth distribution structure of all species in each dataset however, we believe that there is little reason to assume a bias as long as sampling methodology was consistent within each period.

The depth distribution structure has changed from a *Quasi‐nested/random* structure in the 1880s with great overlaps between species distribution ranges, where many ranges reached over a major part of the depth gradient, to a true *Clementsian* structure in recent times, where groups of species with overlapping ranges replaced each other along the depth gradient. The strong positive correlation between the primary RA axis and depth in recent times suggests increased importance of one or several depth‐dependent factors acting in concert. To evaluate a possible bias due to more sampling sites and a higher proportion of deep sites in the 1800s, we permuted samples with the same number of sites as in the recent datasets in the 1880s dataset and repeated the EMS analyses. The outcome of this analysis suggested that a *Clementsian* pattern strongly correlated with depth, as seen in the recent datasets, was an unlikely result using the 1880s dataset. A conspicuous feature of this change is the presence in recent times of many species with distributions shallower than 30 m, 49% in the 1990s and 54% in the 2000s of the total number of species compared to only 16% in the 1880s. The analysis of species present both in old and in recent times shows that for 33% of these species the maximal depth of occurrence had decreased to depths shallower than 30 m in both recent decades, and several species had increased their maximal depth of occurrence at least to the greatest depth of the studied depth interval. Both types of changes likely contributed to the strong *Clementsian* patterns. Furthermore, the substantial differences in taxonomic group composition between decreasing and increasing species support the idea that these changes were nonrandom, as did the EMS analysis. For example, polychaetes and peracarid crustaceans accounted only for some 25% of the species showing decreasing maximal depth and as much as 75% of the increasing species, which contained no gastropods and decapods at all.

A shallower distribution in recent times of many species occurring in both old and recent times may as such be in line with at least three human‐induced scenarios acting in concert, eutrophication‐induced narrowing of the euphotic zone, and hypoxia/anoxia and trawling impacts in the deeper part of the depth interval.

*Eutrophication‐induced narrowing of the euphotic zone*. Middelboe and Sand‐Jensen (2000) reported an estimated decrease in the Secchi depth in the southern Kattegat from ca. 10 m in the 1940s to 5 m in the 1980s. Henriksen (2009) found a negative relationship between N‐inputs and Secchi depth in the Kattegat, and the Secchi depth was ca. 5 m less in the 1980s compared to the 1910s. Secchi depth decreased in the 1900s also in the adjacent Baltic Sea (Sanden & Håkansson, 1996; ca. 5 m in 100 years). The depth penetration of the autotrophic *Zostera marina* in the Kattegat has decreased from at least 20 m in the 1880s (Petersen, 1893) to 10 m or less in recent times (Duarte, 1991; Nielsen, Sand‐Jensen, Borum, & Geertz‐Hansen, 2002). Although the group of species with shallower distribution in recent times contained most large bivalves (Supporting Information Table S3), that is potential microplankton feeders, the magnitude of the decrease in their maximal depth of occurrence was much greater (ca. 20 m), than the change in Secchi depth. Furthermore, the depth interval over which the change occurred was 80–20 m, that is largely deeper than the change in Secchi depth. Thus, there is a poor match of fauna changes with changes in the depth limit of the photic zone.

*Neither does hypoxia/anoxia*, another factor related to eutrophication, offer a plausible cause of the fauna changes. The depth distribution structure (Figures 4 and 5), the changes in maximal depths of occurrence of individual species, and the composition in terms of higher taxonomic groups and traits (Figures 6, 7, 8) are more or less identical in the two recent datasets collected 10 years apart. This could suggest that the system has been in an altered state at least over this period, without much recovery. Unlike the adjacent Baltic Sea where anoxia is widespread in deeper areas, the open Kattegat is a shallow well‐flushed sea area, with only occasional reports of intermittent hypoxia and then with local extent (Conley et al., 2007; Rosenberg, Loo, & Möller, 1992). The 1990s dataset was sampled 10 years after the main hypoxic event in 1988 (Rosenberg et al., 1992), and the 2000s dataset 6 years after the 2002 event (Conley et al., 2007). Recovery time after anoxia‐induced extermination of the fauna in some Swedish coastal areas may be some 5–6 years for most species (e.g., Josefson, Blomqvist, Hansen, Rosenberg, & Rygg, 2009). So, even if the hypoxic events had killed off the fauna in the >30 m zone, there would still be time for complete recovery of most species with shallower distribution today. Furthermore, several of these local hypoxic events, for instance the one in 1988, which occurred close to the halocline at 15–20 m, occurred shallower than 30 m and not so much in the deep, fished zone (Rosenberg et al., 1992). Therefore, it seems highly unlikely that these events have permanently changed the depth distributions as observed in our study.

A remaining possible cause of the fauna changes is adverse *effects of bottom trawling*. Commonly used trawling gears in eastern Kattegat are otter trawls with different ground gears to ensure contact with the sea floor (e.g., Eigaard et al., 2016), thus exerting mechanical disturbance in the surficial bottom sediments, particularly in soft muds (Kaiser et al., 2006). As pointed out by Pommer et al. (2016), chronic trawling has occurred in the deeper parts of the Kattegat for at least 80 years and it is therefore difficult, not to say impossible, to find suitable spatial reference areas suitable to trawling. In our study, we use a temporal reference from the time long before intensive trawling in the Kattegat and we notice substantial changes in the depth distribution of species particularly in the area trawled in recent times. Nowadays, trawling in the Kattegat is intensive on most soft bottoms deeper than 22 m, with peak intensities at 35–50 m on the Swedish side based on data from 2007 to 2009 (Pommer et al., 2016). Intensity measured by the SAR (swept area ratio) for the period 2009–2015 (ICES, 2016) on sites in our study indicates highest impact on sites deeper than ca. 30 m (Figure 3) with peak intensity in fair agreement with Pommer et al. (2016). It may therefore be more than a coincidence that the maximal depth occurrence of many species has moved upward to, or above 30 m, away from the depth zone intensively trawled today.

We find that the composition in terms of traits differs markedly between most species with shallower occurrence than 30 m in recent times and species that have remained within the depth zone deeper than 30 m (Figure 8). A majority of the species in the first mentioned group (green in Figure 8) are emergent or semiemergent and contain most species with large maximal body size in this trait category, traits that make them sensitive to trawling (Kaiser et al., 2000; Supporting Information Table S3 our study). In contrast, the infauna trait group is highly dominated by species remaining in the deep zone fished today (brown in Figure 8), particularly small‐sized infauna. The differences in trait composition between the species with shallower occurrence and species remaining in the fished zone reflect to some extent differences in taxonomic composition between decreasing and increasing species with respect to maximal depth. Large‐bodied emergent/semiemergent species contain most large bivalves, gastropods, and decapods, while small infauna mostly are polychaetes. Of the species category with deeper distribution in recent times, but still occurring in the HFP zone, a majority belonged to *Peracarida* crustaceans and polychaetes. Most species of these groups are small and short‐lived, many polychaetes with a longevity of ca. 2 years and several *Peracarida* crustaceans like the *Cumacea* species even annual. Polychaetes are truly infaunal although several feed at the sediment–water interface. We expect species with these traits to survive in trawled areas (e.g.*,* Kaiser et al., 2000). For instance, the fishing gears are less likely to injure small‐bodied species because of displacement by the pressure wave in front of the gear (Gilkinson, Paulin, Hurley, & Schwinghamer, 1998), and small‐bodied taxa are typically able to recover fast and hence withstand higher levels of chronic trawling.

All *Peracarida* crustaceans “moving” from the fished zone to the unfished zone in one or both of the recent decades were semiemergent and included several species making tubes or pillars protruding above the sediment surface, that is from the genera *Dyopedos*,* Ampelisca*, and *Haploops*. The last mentioned formed extensive tube mats in deeper areas in the pretrawling period (Petersen, 1913) and is now only found occasionally in the Kattegat. For instance in the present study, *Haploops tubicola* occurred at 22 of 57 sites evenly spread in the depth interval of 22–80 m in the 1880s and occurred only at one site at 23 m in the 1990s (Supporting Information Table S2). The disappearance of *Haploops* tube mats in the Kattegat is probably one obvious effect of demersal trawling, because mats, although with local extent, still existed in the 2000s in the Sound (Josefson & Pedersen, 2001) with a similar physical environment but with a trawling ban.

So, the decreases in maximal depth away from the fished zone, and the different species and trait composition of decreasing and species remaining in the fished zone are as argued above, are most easily explained by benthic trawling impacts in recent times. Concurrently, richness in the investigation area corrected for sampling effort did not decrease, and therefore, it seems that the major effect of trawling is changes in spatial distribution of species within the area rather than net species loss.

To conclude, the depth distribution structure of benthic invertebrate fauna in the eastern Kattegat has changed substantially since the 1880s. Many species, including the species likely to suffer most from intensive trawling, have narrower depth distribution intervals in recent times with changed maximal depth limits above and away from the fished zone. Our study using a temporal preimpact reference underpins earlier suggestions that chronic fishing disturbance has changed benthic community structure over wide areas of the shelf seas (e.g., Kaiser et al., 2000). We suggest that the Petersen dataset from the 1880s, despite some taxonomic and methodological caveats, is highly suitable for setting reference conditions for management of benthic species distributions in this sea area.

## CONFLICT OF INTEREST

None declared.

## AUTHORS CONTRIBUTION

AJ and LL gave the idea and conceived the study; AJ wrote the paper; LL, MB, and JR collated the data; AJ and MB analyzed the data. All performed critical reading and contribution to the revisions.

## DATA ACCESSIBILITY

All primary station and fauna data are contained within the paper and its appendix files.

Fishing pressure data from ICES are available from https://odims.ospar.org/.
