# Supplementary material for: Substantial changes in the depth distributions of benthic invertebrates in the eastern Kattegat since the 1880s
Source: Ecol Evol. 2018 Aug 29;8(18):9426–38. doi: 10.1002/ece3.4395 (PMC6194265; doi:10.1002/ece3.4395)
Supplement: Supplementary file 1 [file ECE3-8-9426-s001.docx]

Table S1. Site list with positions, water depths and sampling dates.

| Site id | Latitude N | Longitude E | Depth (meters) | Decade of visit |
| --- | --- | --- | --- | --- |
| 428 | 56,57900 | 12,60006 | 21,0 | 1880 |
| 427 | 56,70569 | 12,42836 | 22,9 | 1880 |
| 413 | 56,26725 | 12,35539 | 26,5 | 1880 |
| 437 | 56,39761 | 12,11967 | 28,4 | 1880 |
| 223 | 56,98956 | 11,85136 | 28,4 | 1880 |
| 426 | 56,83083 | 12,26036 | 28,4 | 1880 |
| 212 | 56,84947 | 11,90178 | 29,3 | 1880 |
| 415 | 56,39789 | 12,36275 | 29,3 | 1880 |
| 416 | 56,51564 | 12,44150 | 29,3 | 1880 |
| 417 | 56,53417 | 12,40578 | 29,3 | 1880 |
| 414 | 56,32172 | 12,32808 | 30,2 | 1880 |
| 431 | 56,69333 | 12,09183 | 30,2 | 1880 |
| 499 | 56,82681 | 12,00258 | 30,2 | 1880 |
| 43 | 57,09978 | 11,71119 | 31,1 | 1880 |
| 217 | 56,88275 | 11,88917 | 31,1 | 1880 |
| 235 | 57,57178 | 11,64925 | 31,1 | 1880 |
| 508 | 56,42897 | 12,23358 | 31,1 | 1880 |
| 419 | 56,60761 | 12,13803 | 32,0 | 1880 |
| 241 | 57,10689 | 12,11492 | 32,9 | 1880 |
| 429 | 56,64889 | 12,36117 | 32,9 | 1880 |
| 497 | 56,93433 | 12,19814 | 34,8 | 1880 |
| 418 | 56,58103 | 12,31286 | 36,6 | 1880 |
| 425 | 56,82078 | 12,17111 | 36,6 | 1880 |
| 498 | 56,88936 | 12,07608 | 37,5 | 1880 |
| 423 | 56,77881 | 11,97108 | 40,3 | 1880 |
| 473 | 57,26217 | 11,81986 | 40,3 | 1880 |
| 33 | 57,51181 | 11,43925 | 42,1 | 1880 |
| 430 | 56,67197 | 12,22886 | 42,1 | 1880 |
| 507 | 56,51103 | 12,20839 | 42,1 | 1880 |
| 319 | 57,05897 | 11,96531 | 43,9 | 1880 |
| 224 | 57,01100 | 11,75319 | 44,8 | 1880 |
| 454 | 57,66117 | 11,23397 | 44,8 | 1880 |
| 31 | 57,54814 | 11,35261 | 45,8 | 1880 |
| 316 | 57,14506 | 11,67497 | 45,8 | 1880 |
| 317 | 57,11003 | 11,66919 | 45,8 | 1880 |
| 424 | 56,78772 | 12,08764 | 45,8 | 1880 |
| 36 | 57,31239 | 11,72642 | 47,6 | 1880 |
| 449 | 57,77047 | 11,09064 | 47,6 | 1880 |
| 320 | 57,02258 | 12,12044 | 49,4 | 1880 |
| 44 | 57,04528 | 11,73428 | 51,2 | 1880 |
| 470 | 57,41894 | 11,67811 | 52,2 | 1880 |
| 239 | 57,24089 | 11,67025 | 53,1 | 1880 |
| 469 | 57,39747 | 11,56578 | 54,9 | 1880 |
| 37 | 57,21533 | 11,91542 | 54,9 | 1880 |
| 240 | 57,14536 | 11,99839 | 54,9 | 1880 |
| 318 | 57,08808 | 11,83983 | 54,9 | 1880 |
| 453 | 57,65217 | 11,35944 | 54,9 | 1880 |
| 466 | 57,48106 | 11,47914 | 54,9 | 1880 |
| 242 | 57,16186 | 11,83825 | 56,7 | 1880 |
| 471 | 57,35742 | 11,71908 | 57,6 | 1880 |
| 250 | 57,17297 | 11,66236 | 58,6 | 1880 |
| 465 | 57,49658 | 11,52639 | 60,4 | 1880 |
| 450 | 57,76797 | 11,30903 | 62,2 | 1880 |
| 472 | 57,33222 | 11,60828 | 67,7 | 1880 |
| 238 | 57,29622 | 11,56419 | 71,4 | 1880 |
| 236 | 57,56278 | 11,53533 | 80,5 | 1880 |
| 475 | 57,20139 | 11,66342 | 80,5 | 1880 |
| N8 | 57,26867 | 12,08333 | 19,0 | 1990, 2000 |
| S5 | 56,31550 | 12,65217 | 19,7 | 1990, 2000 |
| L9 | 56,56500 | 12,72000 | 20,0 | 1990, 2000 |
| N11 | 57,18000 | 12,08333 | 20,0 | 1990, 2000 |
| L4 | 56,61666 | 12,64067 | 21,0 | 1990, 2000 |
| N9 | 57,22500 | 12,07417 | 21,0 | 1990, 2000 |
| N15 | 56,72167 | 12,44167 | 23,0 | 1990, 2000 |
| ÖVF 1:3 | 56,20117 | 12,47450 | 23,0 | 1990, 2000 |
| N13 | 57,13667 | 12,10667 | 24,0 | 1990, 2000 |
| N7 | 57,30333 | 11,98833 | 26,0 | 1990, 2000 |
| Danafjord | 57,67000 | 11,69000 | 26,5 | 1990, 2000 |
| N6 | 57,36000 | 12,02917 | 27,0 | 1990, 2000 |
| ÖVF 2:3 | 56,02833 | 12,68667 | 29,0 | 1990, 2000 |
| N14 | 56,94000 | 12,21167 | 31,0 | 1990, 2000 |
| N12 | 57,18000 | 12,01833 | 48,0 | 1990, 2000 |
| N10 | 57,12000 | 12,01967 | 50,0 | 1990, 2000 |
| ANHOLT | 56,66667 | 12,11667 | 53,5 | 1990, 2000 |
| FLADEN | 57,19117 | 11,66667 | 67,0 | 1990, 2000 |
| SWVIN | 57,55000 | 11,52500 | 76,0 | 1990, 2000 |
